# Doenças infecciosas e parasitárias no Brasil de 2010 a 2017: aspectos para vigilância em saúde

**DOI:** 10.26633/RPSP.2020.10

**Published:** 2020-02-10

**Authors:** Helen Paredes de Souza, Wanessa Tenório Gonçalves Holanda de Oliveira, Jefferson Pereira Caldas dos Santos, João Paulo Toledo, Isis Polianna Silva Ferreira, Suely Nilsa Guedes de Sousa Esashika, Tatiane Fernandes Portal de Lima, Amanda de Sousa Delácio

**Affiliations:** 1 Instituto Nacional de Câncer José Alencar Gomes da Silva (INCA) Instituto Nacional de Câncer José Alencar Gomes da Silva (INCA) Rio de Janeiro (RJ) Brasil Instituto Nacional de Câncer José Alencar Gomes da Silva (INCA), Rio de Janeiro (RJ), Brasil.; 2 Ministério da Saúde Departamento de Imunização e Doenças Transmissíveis Brasília (DF) Brasil Ministério da Saúde, Departamento de Imunização e Doenças Transmissíveis, Brasília (DF), Brasil.; 3 Fundação Oswaldo Cruz (FIOCRUZ) Centro de Inovação em Biodiversidade e Saúde Rio de Janeiro (RJ) Brasil Fundação Oswaldo Cruz (FIOCRUZ), Centro de Inovação em Biodiversidade e Saúde, Rio de Janeiro (RJ), Brasil.; 4 Organização Pan-Americana da Saúde (OPAS) Consultor em Doenças Infecciosas Brasília (DF) Brasil Organização Pan-Americana da Saúde (OPAS), Consultor em Doenças Infecciosas, Brasília (DF), Brasil.; 5 Ministério da Saúde Departamento de Gestão e Incorporação de Tecnologias e Inovação em Saúde Brasília (DF) Brasil Ministério da Saúde, Departamento de Gestão e Incorporação de Tecnologias e Inovação em Saúde, Brasília (DF), Brasil.; 6 Ministério da Saúde Departamento de Promoção da Saúde Brasília (DF) Brasil Ministério da Saúde, Departamento de Promoção da Saúde, Brasília (DF), Brasil.

**Keywords:** Doenças transmissíveis, fatores socioeconômicos, análise espacial, Brasil, Communicable diseases, socioeconomic factors, spatial analysis, Brazil, Enfermedades transmisibles, factores socioeconómicos, análisis espacial, Brasil

## Abstract

**Objetivo.:**

Apresentar um método para identificar áreas críticas relativas a doenças infecciosas e parasitárias selecionadas para fins de vigilância em saúde, analisando a sua associação a indicadores de pobreza no Brasil.

**Métodos.:**

Foram mapeadas as taxas de incidência de dengue, doença de Chagas aguda, esquistossomose, hanseníase, hepatite A, leishmaniose tegumentar, leishmaniose visceral, leptospirose, malária e tuberculose. Foram realizadas análises para os anos de 2010 a 2017 a partir de um indicador síntese, calculado como a média dos coeficientes médios de incidência para cada agravo normalizada pela média e desvio padrão durante o período analisado. A estimativa da base populacional foi de 2014. Os coeficientes calculados foram estratificados para classificação dos municípios em criticidade muito alta, alta, média, baixa ou muito baixa conforme cada doença. Também foram selecionados indicadores de diferentes dimensões que expressassem desigualdades socioeconômicas e segregação espacial nos municípios brasileiros, sendo testada a sua associação às doenças em estudo.

**Resultados.:**

O indicador mostrou que 40,5% dos municípios brasileiros apresentam alta criticidade, sobretudo nas regiões Norte, parte do Nordeste e Centro-Oeste. Os indicadores “proporção de pobreza”, “lixo no entorno”, “esgoto no entorno” e “famílias chefiadas por mulheres” podem aumentar a chance de a localidade apresentar maior criticidade para as doenças. O indicador “esgoto adequado” pode ser considerado potencial fator de proteção.

**Conclusões.:**

A técnica utilizada foi adequada para orientar ações de vigilância no país e permite a articulação entre vigilâncias locais e demais setores para contornar os problemas de saúde causados por doenças infecciosas e parasitárias e fatores relacionados.

## Abstract (en)

**Objective.:**

To present a method to identify critical areas for selected infectious and parasitic diseases for the purpose of health surveillance and to analyze the association between these critical areas and poverty indicators in Brazil.

**Method.:**

The following incidence rates were mapped: dengue, acute Chagas disease, schistosomiasis, Hansen's disease, hepatitis A, cutaneous leishmaniasis, visceral leishmaniasis, leptospirosis, malaria, and tuberculosis. The analyses were performed for the period from 2010 to 2017 based on a synthetic indicator calculated as the mean of mean incidence coefficients for each disorder, normalized by the mean and standard deviation during the period of analysis. A 2014 population estimate was used. The calculated coefficients were stratified for classification of municipalities into very high, high, medium, low, or very low criticality according to each disorder. Indicators expressing several socioeconomic dimensions and space segregation in Brazilian municipalities were also selected and tested regarding their association with the transmission of the diseases under study.

**Results.:**

The indicator showed that 40.5% of Brazilian municipalities had high criticality for the diseases of interest, especially in the North, parts of the Northeast, and Midwest. Indicators “proportion of poverty,” “garbage in surroundings,” and “families headed by women” increased the chance of higher criticality for the diseases. The indicator “adequate sewer system” was a potential protection factor.

**Conclusions.:**

The technique used was adequate to guide surveillance actions in the country and allows articulation between local surveillance efforts and other sectors to resolve health problems caused by infectious and parasitic diseases and associated factors.

## RESUMEN

**Objetivo.:**

Presentar un método para identificar áreas críticas relacionadas con ciertas enfermedades infecciosas y parasitarias con fines de vigilancia sanitaria y analizar su asociación con los indicadores de pobreza en Brasil.

**Métodos.:**

Se cartografiaron las tasas de incidencia de dengue, enfermedad de Chagas aguda, esquistosomiasis, lepra, hepatitis A, leishmaniasis cutánea, leishmaniasis visceral, leptospirosis, malaria y tuberculosis. Se efectuaron análisis para los años 2010 a 2017 a partir de un indicador de síntesis, calculado como el promedio de los coeficientes de incidencia promedio para cada enfermedad, normalizado por la media y la desviación estándar durante el período analizado. La base de población estimada fue la de 2014. Los coeficientes calculados se estratificaron para clasificar los municipios según presentaran una situación crítica muy alta, alta, media, baja o muy baja para cada enfermedad. Se seleccionaron también indicadores de diferentes dimensiones que expresaran las desigualdades socioeconómicas y la segregación espacial en los municipios brasileños, y se evaluó su asociación con las enfermedades estudiadas.

**Resultados.:**

El indicador demostró que el 40,5% de los municipios brasileños presentan una situación crítica alta, en especial en las regiones Norte y Centro-oeste y parte del Nordeste. Los indicadores "proporción de pobreza", "basura en los alrededores", "aguas servidas en los alrededores" y "familias encabezadas por mujeres" pueden aumentar la posibilidad de que la localidad presente una situación más crítica para las enfermedades. El indicador "red cloacal adecuada" puede considerarse un potencial factor de protección.

**Conclusiones.:**

La técnica utilizada fue adecuada para orientar las acciones de vigilancia sanitaria en el país y permite la articulación entre la vigilancia local y otros sectores para evitar los problemas de salud causados por las enfermedades infecciosas y parasitarias y los factores relacionados.

As doenças infecciosas e parasitárias têm grande importância para a saúde pública por estarem diretamente associadas à pobreza e a condições de vida inadequadas. O padrão de distribuição espacial de sua ocorrência pode ser utilizado como *proxy* das condições de desenvolvimento de áreas geograficamente delimitadas, relacionando-se aos indicadores epidemiológicos e de qualidade de vida das populações. No Brasil, apesar do declínio da morbimortalidade desde a década de 1960, essas doenças persistem (1-3), num cenário de transição epidemiológica e demográfica marcado pela predominância concomitante de doenças transmissíveis e crônico-degenerativas, pelo recrudescimento de algumas doenças já em vias de controle e eliminação e pelo contraste no quadro epidemiológico entre diferentes regiões do país (1-3).

Esse cenário multifacetado, dentro de um contexto social urbano diverso, torna complexo o trabalho da vigilância em saúde em seu papel primordial de coleta, consolidação, avaliação e disseminação de informações para subsidiar a tomada de decisão, exigindo constantes inovações, sobretudo na forma de articular as diversas realidades contidas nas conjunturas das vigilâncias locais. Nesse contexto, a identificação de áreas críticas para doenças infecciosas e parasitárias no Brasil e o conhecimento sobre a sua relação com indicadores socioeconômicos são de fundamental importância para alinhar as ações de vigilância nos âmbitos local e nacional, fornecendo subsídios para o estabelecimento de medidas assertivas de controle, planejamento e intervenção, bem como para articular ações intersetoriais de mitigação das causas determinantes desses adoecimentos.

O uso de ferramentas de análise espacial em saúde pública ajuda a fomentar a discussão sobre o espaço e a heterogeneidade dos fenômenos populacionais nele distribuídos, auxiliando no reconhecimento de áreas com características socioambientais mais semelhantes entre si e identificando locais de maior vulnerabilidades e risco à saúde (4, 5). Em razão da importância da distinção de áreas prioritárias para ações em saúde pública, o presente estudo tem como objetivo apresentar um método simples, desenvolvido para identificar áreas críticas quanto à incidência de doenças infecciosas e parasitárias selecionadas, analisando a associação ecológica entre a ocorrência dessas doenças e indicadores de pobreza no Brasil.

## MATERIAIS E MÉTODOS

Foi realizado um estudo observacional, analítico e ecológico que utilizou informações referentes a adoecimento por algumas doenças infecciosas e parasitárias relacionadas à pobreza em todos os municípios brasileiros. O Brasil é composto por cinco regiões, divididas em 27 unidades da federação, com 5 570 municípios (unidade de análise).

Foram selecionadas doenças infecciosas e parasitárias epidemiologicamente relevantes enquanto problema de saúde pública no Brasil, de notificação compulsória (6), com dados publicamente disponíveis no DATASUS relativos ao período de estudo. Os agravos selecionados, de acordo com a 10^a^ Classificação Internacional de Doenças (CID-10, Capítulo I, código de A00 a B99), foram: dengue (A90 a A99); doença de Chagas aguda (B57.0 a B57.2); esquistossomose (B65); hanseníase (A30); hepatite A (B15); leishmaniose tegumentar (B55.1 e B55.2); leishmaniose visceral (B55.0); leptospirose (A27); malária (B50 a B54); e tuberculose (A15 a A19).

Optou-se pela utilização apenas de dados secundários de domínio público, sem cruzamento de informações com outras fontes de dados. Os dados foram solicitados ao Ministério da Saúde via Sistema Eletrônico do Serviço de Informações ao Cidadão (e-SIC) em 29 de outubro de 2018, sob protocolo número 25820006870201805. Foram solicitados e fornecidos os números de casos novos confirmados notificados ao Sistema de Informação de Agravos de Notificação (SINAN) por município de residência, de 2010 a 2017 separadamente, para todas as doenças de interesse. Os dados de esquistossomose são oriundos de notificação ao SINAN e de busca ativa de casos notificados ao Sistema de Informação da Esquistossomose (SISPCE). Os dados de malária são provenientes, além do SINAN, do Sistema de Informação de Vigilância Epidemiológica (SIVEP-malária), específico para notificações da região amazônica.

As análises foram realizadas para os anos de 2010 a 2017, obtendo-se os coeficientes médios de incidência de cada uma das causas de adoecimento isoladamente, ajustados pelo método bayesiano empírico local, com o objetivo de minimizar as flutuações decorrentes do pequeno número de óbitos e população em algumas localidades (7). Em seguida, esses valores de coeficiente foram normalizados separadamente pela média e desvio padrão do coeficiente durante o período analisado. A partir dos valores normalizados foi calculada uma média, que correspondeu ao indicador síntese. Dessa maneira, os coeficientes, assim como o indicador síntese, variaram de 0 a 1. A normalização se faz necessária para retirar o efeito da magnitude diferenciada que cada agravo possui em relação à incidência. A estimativa da base populacional foi a do ano de 2014, denominador usado para o cálculo dos coeficientes médios a partir de dados estimados do Instituto Brasileiro de Geografia e Estatística (IBGE).

Os coeficientes assim calculados foram estratificados para classificação dos municípios em criticidade muito alta, alta, média, baixa ou muito baixa, conforme cada causa de adoecimento. Os valores atribuídos aos municípios a partir da classificação segundo indicador síntese também tiveram a mesma estratificação.

A malha cartográfica de municípios utilizada neste estudo, referente ao ano de 2015, foi obtida do IBGE. Os mapas e as análises espaciais foram realizados na plataforma *ArcGis*, versão 10.6.

### Análise estatística

Todos os dados utilizados neste estudo são de domínio público e acesso livre (8). Foram selecionados indicadores de diferentes dimensões que pudessem expressar desigualdades socioeconômicas e segregação espacial nos municípios brasileiros, com possibilidade de associação à transmissão de doenças infecciosas e parasitárias. A tabela 1 mostra as variáveis utilizadas e a forma como os indicadores foram operacionalizados. Os dados censitários utilizados foram aqueles referentes ao censo demográfico de 2010, realizado pelo IBGE para caracterizações socioeconômicas, agregados por município.

Para a identificação de variáveis socioambientais associadas somente à situação de criticidade dos municípios, empregou-se um modelo de regressão linear generalizada com distribuição gama e função de ligação log (GLM Gamma Log). Esse modelo leva em consideração o componente espacial na modelagem. Antes da modelagem, realizou-se um teste de multicolinearidade para o fator de inflação de variância (v*ariance inflation factor*, VIF) entre as variáveis explicativas, sendo selecionadas aquelas que apresentaram VIF < 10. Não foram construídos modelos para cada agravo isolado, apenas para o índice de criticidade de cada município frente aos valores dos indicadores socioambientais que cada município apresentou.

Por tratar-se de estudo com base populacional, a técnica utilizada não tem a finalidade de testar hipóteses; apenas pretende descrever as variáveis associadas à classificação de criticidade atribuída aos municípios. As análises estatísticas foram realizadas no pacote estatístico *R* versão 2.11.1 (*R Development Core Team* 2012).

**TABELA 1.** Variáveis utilizadas para modelagem estatística e operacionalização dos indicadores de pobreza em municípios brasileiros

Dimensões e variáveis	Operacionalização dos indicadores
Saneamento ambiental	
Água	Proporção de domicílios com serviço de abastecimento de água adequado entre o total de domicílios ligados à rede geral
Esgoto no entorno	Proporção de domicílios com esgotamento a céu aberto entre o total de domicílios
Esgoto adequado	Proporção de domicílios com serviço de esgoto adequado ligados à rede geral ou pluvial
Lixo no entorno	Proporção de domicílios com lixo acumulado em seu entorno entre o total de domicílios
Coleta de lixo adequada	Proporção de domicílios com serviço de lixo adequado, coletado por serviço público de limpeza ou colocado em caçamba de serviço de limpeza
Habitação	
Casa própria	Proporção de domicílios particulares permanentes próprios e quitados ou em aquisição entre o total de domicílios
Educação	
Baixa escolaridade	Proporção de pessoas residentes com escolaridade desde sem instrução a ensino fundamental incompleto entre o total de residentes
Renda	
Proporção de pobreza	Proporção de pessoas responsáveis pelo domicílio com rendimento nominal mensal de até 1 salário mínimo, incluindo as que não têm renda
Famílias chefiadas por mulheres	Proporção de pessoas responsáveis do sexo feminino entre o total de pessoas responsáveis
Densidade de pobreza	Número de pessoas responsáveis pelo domicílio com renda até 1 salário mínimo por área ocupada em quilômetros quadrados

***Fonte:*** Instituto Brasileiro de Geografia e Estatística (IBGE) - Censo 2010.

## RESULTADOS

Entre os anos de 2010 e 2017 foram notificados 10 578 337 casos de adoecimento pelas causas consideradas nesta investigação, correspondendo a uma taxa bruta de 5 218,72 casos por 100 000 habitantes. A dengue foi responsável por quase 70% dos adoecimentos.

**FIGURA 1.**
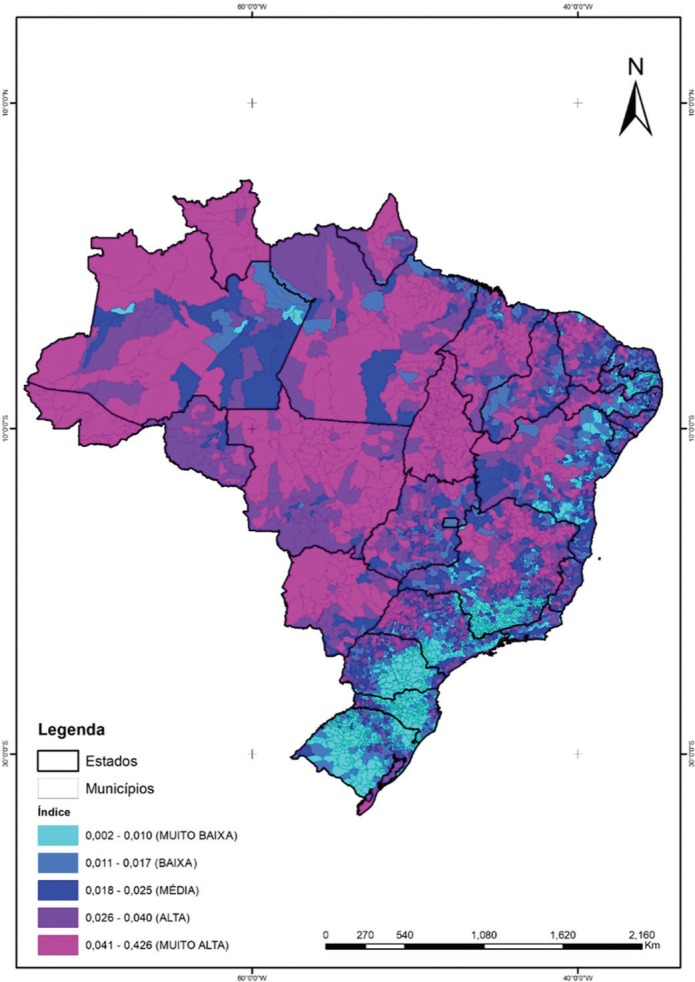
Classificação dos municípios brasileiros segundo indicador sintético de criticidade por doenças infecciosas e parasitárias, 2010 a 2017

A figura 1, que mostra a classificação dos municípios brasileiros segundo indicador sintético de criticidade de acordo com a incidência média de doenças infecciosas e parasitárias, aponta que 41% dos municípios foram classificados como sendo de criticidade baixa ou muito baixa para doenças infecciosas e parasitárias relacionadas à pobreza; 18,5% apresentam criticidade intermediária, enquanto 40,5% das localidades apresentam criticidade alta ou muito alta. Observa-se ainda que os municípios do Sul, Sudeste e Nordeste apresentaram, em sua maioria, baixa ou muita baixa situação de criticidade, enquanto os do Norte e Centro-Oeste foram classificados com criticidade intermediária, alta ou muito alta.

***Fonte:*** Ministério da Saúde, Sistema de Informação de Agravos de Notificação (SINAN), 2010 a 2017.

A tabela 2 apresenta os indicadores que mostraram associação estatisticamente significativa com o indicador sintético de criticidade no modelo de regressão linear generalizada com distribuição gama e função de ligação log. Quatro indicadores apresentaram associação direta com o indicador síntese: esgoto no entorno, lixo no entorno, proporção de pobreza e famílias chefiadas por mulheres, podendo ser considerados possíveis fatores que aumentam a probabilidade de a localidade apresentar maior criticidade para doenças infecciosas e parasitárias. Já o indicador “esgoto adequado” mostrou associação inversa, podendo ser considerado fator de proteção: à medida que sua proporção aumenta, diminui a probabilidade de as áreas apresentarem alta criticidade para doenças infecciosas e parasitárias. O modelo apresentou ajuste satisfatório tanto pela análise dos resíduos quanto pelo critério de informação de Akaike (AIC).

**FIGURA 2.**
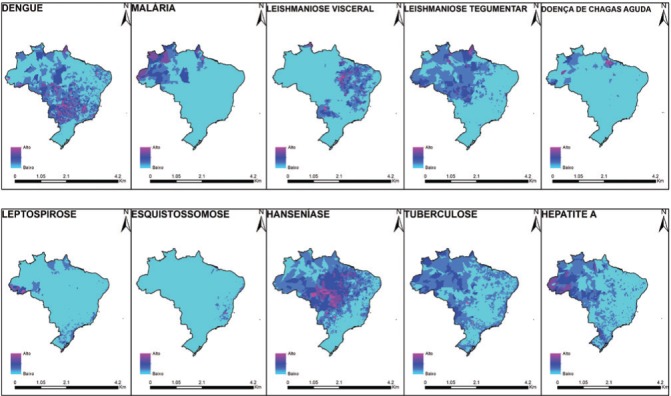
Coeficientes médios de incidência de doenças infecciosas e parasitárias selecionadas ajustados pelo método bayesiano empírico local nos municípios brasileiros, 2010 a 2017

A figura 2 mostra a taxa de incidência média de cada agravo separadamente, ajustada pelo método bayesiano empírico local. Observa-se que as incidências de dengue, hanseníase, tuberculose, hepatite e leishmaniose tegumentar têm um padrão de maior difusão pelos municípios brasileiros, com maior expressão nas regiões Centro-Oeste e Norte. A incidência de leishmaniose visceral é mais expressiva no Nordeste e no Centro-Oeste, assim como a de esquistossomose é mais expressiva no Sudeste e no Nordeste. Já a malária e a doença de Chagas apresentam maior magnitude no Norte, e a leptospirose no Norte e no Sul do país.

**TABELA 2.** Modelagem^a^ do indicador sintético de criticidade por doenças infecciosas e parasitárias relacionadas à pobreza e variáveis socioeconômicas e ambientais, Brasil, 2010 a 2017

Indicadores	*B*^a^	P
Baixa escolaridade	0,998	0,36
Esgoto adequado	0,996	<0,01
Esgoto no entorno	1,015	<0,01
Lixo no entorno	1,009	<0,01
Proporção de pobreza	1,003	<0,01
Famílias chefiadas por mulheres	1,008	<0,01

***Fonte:*** Instituto Brasileiro de Geografia e estatística (IBGE), Censo 2010.

a GLM Gamma log.

***Fonte:*** Ministério da Saúde, Sistema de Informação de Agravos de Notificação (SINAN), 2010 a 2017.

## DISCUSSÃO

Os achados do presente estudo apontam que há um gradiente de concentração de maiores incidências de doenças infecciosas e parasitárias, isoladamente ou em conjunto, principalmente nas regiões Norte, Centro-Oeste e sub-região meio-norte do Nordeste do país, diminuindo em direção ao Sul e ao litoral leste. Essas incidências aumentadas estão associadas a piores condições de vida da população.

A conformação revelada neste estudo pode ser reflexo da ocupação e do perfil socioambiental historicamente construídos no território brasileiro. Ao longo de grande parte da história econômica do país, o desenvolvimento enfocou atividades específicas voltadas para exportação, sem preocupação com a integração dos núcleos de desenvolvimento, produzindo complexos econômicos diversos não necessariamente coordenados, concentrados nos grandes centros metropolitanos, nas zonas litorâneas do Nordeste e nas regiões Sul e Sudeste. Da mesma forma, com o avanço da fronteira agrícola para as regiões Centro-Oeste e sul da região Norte, alguns centros urbanos emergiram nessas regiões, sem, contudo, terem uma integração completa com o tecido urbano nacional (9). Mesmo com a redução dos patamares médios da pobreza, os índices de desigualdade permanecem elevados e se expressam de forma significativa em determinados grupos sociais mais vulnerabilizados (9). Considerando que as condições de vida têm impacto direto sobre as condições de saúde da população, o distanciamento geográfico-sócio-ambiental também se reflete na distribuição espacial das doenças infecciosas e parasitárias no Brasil, conforme aponta o mapa do indicador síntese, que considera o conjunto das doenças infecciosas e parasitárias para fins de vigilância, evidenciando a transição epidemiológica polarizada vivenciada no Brasil (1, 2).

Assim, há também uma regionalização em relação às doenças infecciosas e parasitárias consideradas individualmente na investigação, que apresentam diferentes padrões de distribuição espacial. Observa-se que doenças essencialmente rurais, tais como malária e doença de Chagas, se localizam mais expressamente nos municípios do interior das regiões Norte e Nordeste, onde se conservam características geográficas mais próximas a ambientes rurais (3, 10). Doenças consideradas reemergentes, ou também aquelas infecciosas em declínio no Brasil, tais como dengue, tuberculose e hanseníase, apresentam padrão difuso em todo o território nacional, característica esperada quando o adoecimento é associado aos processos próprios da urbanização. A leishmaniose visceral e a esquistossomose apresentam padrão de transição geográfica em função do contínuo processo de urbanização da população e da replicação de condições ambientais favorecedoras da reprodução e expansão do ciclo de transmissão dessas doenças (3, 10, 11).

O mapa síntese também aponta muitos municípios da região Nordeste classificados como área de baixa criticidade durante o período analisado. A subnotificação de casos de adoecimento, frequentemente reportada à região Nordeste, pode ter influenciado essa classificação (12, 13). Porém, a melhoria de indicadores de habitação observada no Nordeste nos últimos anos, ainda que insuficiente, impacta positivamente a situação geral de saúde daquela população. Um importante *proxy* dessa melhoria é o programa governamental de habitação intitulado Minha casa, minha vida, que consiste na aquisição de terreno e construção de habitações para destinação a famílias cadastradas no programa, priorizando as famílias de baixa renda. Em 2009 e 2010, o programa contratou cerca de 1 milhão de unidades habitacionais (14, 15). O programa previa instalações sanitárias nas moradias, o que pode impactar diretamente a incidência de doenças transmissíveis e parasitárias.

Na região Norte, a migração é um aspecto que não pode ser ignorado na discussão da criticidade por doenças infecciosas e parasitárias. Esse é um fator que vem agravando cenários socioeconômicos, ambientais e demográficos já desfavoráveis em muitos municípios brasileiros de fronteira, principalmente com a Bolívia, Colômbia e Venezuela, países que vêm enfrentando problemas graves de violência e crise social (16). Esses grupos populacionais migram com perspectivas de melhores condições de vida, frustradas na maioria dos casos, sobretudo para populações que, por razões historicamente determinadas, já se encontravam à margem dos processos produtivos em seus países de origem. Além de forte incremento populacional, o aprofundamento de desigualdades no território, bem como a aceleração de processos de urbanização informal e precária, são desdobramentos prováveis dessa situação. Em consequência, o aumento na demanda por serviços de saúde e alterações nos padrões de adoecimento e morte da região são problemas que poderão ser observados. Autores afirmam que migrantes são mais vulneráveis, principalmente a adoecimentos por causas infecciosas e parasitárias, o que deve estar relacionado às condições adversas de vida (17-20).

O achado através da modelagem estatística que aponta associações diretas entre piores condições de saneamento ambiental e altas taxas de adoecimento por doenças infecciosas e parasitárias corrobora os achados de outras investigações (21, 22). Johansen et al. (23) investigaram a distribuição espacial dos casos de dengue em uma área urbana do estado de São Paulo, Brasil, e encontraram associação entre a taxa de incidência de dengue e variáveis sociodemográficas tais como proporção de domicílios com renda *per capita* de até 3 salários mínimos, proporção de pessoas não brancas e proporção de domicílios não proprietários, sugerindo ser a dengue socialmente condicionada (23). O estudo de Ceccon et al. (24), que buscou analisar associações entre mortalidade por tuberculose e indicadores sociodemográficos e de saúde nas capitais dos estados brasileiros e no Distrito Federal, também aponta que o problema é maior em capitais com maior desigualdade de renda associada à migração, à pobreza entre negros e ao coeficiente de coinfecção HIV e tuberculose.

Os indicadores “proporção de pobreza” e “domicílio chefiados por mulheres” foram utilizados como *proxy* de condições socioeconômicas desfavoráveis relativas a todos os moradores do domicílio. Este último é visto pelo viés de gênero, através do qual se evidenciam as diferenças de níveis de pobreza entre os domicílios chefiados por mulheres e aqueles chefiados por homens ou casais (25). Aproximando-se do fenômeno conhecido como feminização da pobreza, caracterizado pelo empobrecimento progressivo das condições de vida das mulheres e exclusão social, o indicador reflete as condições de subocupação vivenciadas pelas mulheres devido, principalmente, à exigência de cuidados domésticos e com os filhos, falta de tempo para capacitação profissional e necessidade de ingressar no mercado de trabalho (26). Sem aprofundar as questões de gênero inerentes ao tema, os moradores desses domicílios também estão mais propícios a adoecimento por doenças infecciosas e parasitárias. Fretas et al. (27) concluíram em seu trabalho sobre o perfil sociodemográfico das mulheres cujos filhos nasceram com síndrome congênita da Zika que a epidemia afetou preferencialmente mulheres de baixa renda e classe social mais baixa, sendo determinada pelas desigualdades sociais no Brasil. Cabe ressaltar que a feminização da pobreza tem determinantes estruturais de desigualdade de inserções e acessos (26) que precisam ser discutidos e aprofundados, a fim de permitir a implementação das necessárias políticas públicas infraestruturais.

É inquestionável que muitos outros aspectos sociais, econômicos e ambientais não contemplados neste estudo determinam, em última análise, a situação de saúde de uma população, sobretudo para aqueles sobre os quais incidem as maiores iniquidades estruturais históricas, quando os riscos de adoecimentos estão distribuídos de forma desigual influenciados por aspectos como sexo, cor de pele, faixa etária (28). Além disso, não se pode desconsiderar a história natural de cada uma das doenças analisadas nesta investigação nem as dinâmicas de transmissão dessas doenças, visto que determinam perfis e impactam de formas distintas as condições de saúde das populações onde incidem, requerendo ações específicas de vigilância e de prevenção.

É importante ressaltar que os resultados desta investigação devem ser analisados com cautela, uma vez que o estudo apresenta limitações. Apesar dos inegáveis avanços na qualidade dos dados secundários ao longo dos anos, ainda são necessárias melhorias. Problemas como subnotificação podem ter produzido resultados enviesados (12, 13). Ressalta-se que a notificação dos casos, de responsabilidade dos profissionais de saúde que os identificaram, ainda que suspeita, é a base da produção de informações confiáveis a respeito do perfil de saúde da população, fundamental para o exercício da vigilância em saúde, sobretudo no que tange a sistematização, consolidação, análise e disseminação de dados, planejamento e implementação de políticas públicas para a proteção, prevenção e controle de riscos, agravos e doenças, bem como para a promoção da saúde, além de desenvolvimento de pesquisas científicas, muitas vezes realizadas a partir de sistemas de informação de domínio público. Também é importante destacar que outras doenças infecciosas e parasitárias de menor magnitude, mas de grande impacto na saúde pública no Brasil, que não compuseram o indicador em função da escassez de dados, podem ter implicado em viés de classificação dos municípios segundo indicador sintético de criticidade utilizado neste estudo.

Outra limitação pode ser atribuída à escolha do município como unidade de análise utilizada na investigação. As características do território municipal abarcam, via de regra, heterogeneidades principalmente nos aspectos econômicos e ambientais. Sabe-se que unidades territoriais menores permitem a identificação de aglomerados populacionais cujas condições de vida são mais semelhantes. A operacionalização das variáveis também pode ter produzido resultados equivocados, uma vez que o processo de categorização traz perda de informações inerentes à técnica (29). Além disso, os dados socioambientais são provenientes do último censo demográfico realizado pelo IBGE, já considerado antigo, uma vez que se refere ao ano de 2010. Deve-se levar em conta também a limitação inerente ao estabelecimento de relações causais dos estudos ecológicos.

Para além das limitações, o presente estudo mostrou que a espacialização de doenças de alta magnitude para a realidade brasileira evidencia os contrastes territoriais de condições de saúde associados a fatores determinantes de adoecimentos. A técnica utilizada para identificação de áreas críticas também se mostrou adequada no sentido de orientar as ações de vigilância no âmbito nacional no que tange a necessidade de maior articulação com as vigilâncias locais e demais setores, para implementação de ações conjuntas que possam dirimir os problemas de saúde causados por doenças infecciosas e parasitárias e outros fatores a elas relacionadas (30).

A problemática das doenças infecciosas e suas relações com condições de vida é antiga, e os caminhos apontados para avanços também já são velhos conhecidos. Sabe-se que o comportamento das doenças infecciosas e parasitárias serve como um indicador de desenvolvimento de uma dada região; e que sua magnitude deve servir como norteador da formulação de políticas públicas não apenas no setor saúde, fundamentado em um modelo de atenção mais inclusivo, mas também em práticas intersetoriais de habitação, saneamento, educação e demais serviços que, em conjunto, poderão diminuir iniquidades sociais e produzir melhorias nas condições de vida e saúde das populações.

### Contribuição dos autores.

HPS concebeu o trabalho e participou da coleta, sistematização, análise dos dados e redação; JPCS contribuiu na confecção dos mapas e análise; ASD, TFPL, JPT e SNGSE fizeram análise e redação; IPSF contribuiu para a concepção do trabalho, análise e redação; WTGHO participou da concepção, coleta, sistematização e análise. Todos revisaram e aprovaram a versão a ser publicada.

### Declaração.

As opiniões expressas no manuscrito são de responsabilidade exclusiva dos autores e não refletem necessariamente a opinião ou política da *RPSP/PAJPH* ou da Organização Pan-Americana da Saúde (OPAS).
